# Prevalence and molecular characterization of *Giardia duodenalis* in dairy cattle in Central Inner Mongolia, Northern China

**DOI:** 10.1038/s41598-023-40987-9

**Published:** 2023-08-26

**Authors:** Li Zhao, Zhan-Sheng Zhang, Wen-Xiong Han, Bo Yang, Hai-Liang Chai, Ming-Yuan Wang, Yan Wang, Shan Zhang, Wei-Hong Zhao, Yi-Min Ma, Yong-Jie Zhan, Li-Feng Wang, Yu-Lin Ding, Jin-Ling Wang, Yong-Hong Liu

**Affiliations:** 1 College of Veterinary Medicine, Inner Mongolia Agricultural University, Hohhot, China; 2 Key Laboratory of Clinical Diagnosis and Treatment Technology in Animal Disease, Ministry of Agriculture and Rural Affairs, Hohhot, China; 3 Inner Mongolia Saikexing Reproductive Biotechnology (Group) Co., Ltd., Hohhot, China; 4 Animal Disease Control Center of Ordos, Ordos, China

**Keywords:** Parasite biology, Parasite evolution, Parasite genetics

## Abstract

*Giardia duodenalis* is a gastrointestinal protozoan ubiquitous in nature. It is a confirmed zoonotic pathogen, and cattle are considered a source of giardiasis outbreaks in humans. This study aimed to evaluate the prevalence and multilocus genotype (MLG) of *G. duodenalis* in dairy cattle in Central Inner Mongolia. This study was based on the small subunit ribosomal RNA (SSU rRNA), glutamate dehydrogenase (*gdh*), triosephosphate isomerase (*tpi*), and beta-giardin (*bg*) genes of *G. duodenalis*. DNA extraction, polymerase chain reaction (PCR), and sequence analysis were performed on 505 dairy cattle fecal samples collected in 2021 from six sampling sites and four age groups in Central Inner Mongolia to determine the prevalence and MLG distribution of *G. duodenalis*. The PCR results of *SSU rRNA* revealed that the overall prevalence of *G. duodenalis* was 29.5% (149/505) and that the overall prevalence of the diarrhea and nondiarrhea samples was 31.5% (46/146) and 28.5% (103/359), respectively; the difference was not significant (p > 0.05). *SSU rRNA* sequence analysis revealed that *G. duodenalis* assemblage E (91.1%, 133/146) was primarily detected and that assemblage A (8.9%, 13/146) was detected in 13 samples. The *G. duodenalis*—positive samples were PCR amplified and sequenced for *gdh*, *tpi*, and *bg*, from which 38, 47, and 70 amplified sequences were obtained, respectively. A combination of *G. duodenalis* assemblages A and E were detected in seven samples. Multilocus genotyping yielded 25 different assemblage E MLGs, which formed six subgroups. To the best of our knowledge, this is the first report regarding *G. duodenalis* infection in dairy cattle in Inner Mongolia, China. This study revealed that Inner Mongolian cattle pose a risk of giardiasis transmission to humans and that the distribution of local cattle *G. duodenalis* assemblage E MLGs is diverse. The findings of this study can bridge the knowledge gap in the molecular epidemiological investigation of giardiasis in Central Inner Mongolia.

## Introduction

*Giardia duodenalis*, also known as *Giardia lamblia* or *Giardia intestinalis*, was first described in 1681^1,2^ and is a group of ubiquitous pathogenic gastrointestinal protozoans^3^. The hosts include humans, companion animals, livestock, and wildlife^4^. Human waterborne protozoan parasitic outbreaks worldwide has increased from 325 in an almost 100-year period^5^ to 199 between 2004 and 2010^6^ and at least 381 outbreaks between 2011 and 2016^7^. In the latter two records, *Giardia* was confirmed as the pathogen in 70 (35.2%)^6^ and 141 (37%) cases^7^, respectively*.* It is estimated that 8 × 10^8^ global cases of giardiasis occur annually^2^. Giardiasis is a notifiable disease by the Centers for Disease Control and Prevention in the USA^2^. Cattle are considered the source of waterborne giardiasis outbreaks in humans^4^. Calves are more susceptible to acute infections caused by *G. duodenalis* and adult cattle often do not exhibit clinical signs and tend to have mild *G. duodenalis* infections*.* However, adult animals help maintain persistent infections in cattle and environmental contamination, thereby leading to the spread of giardiasis^8^.

Based on genetic studies using molecular typing techniques, *G. duodenalis* was classified into at least eight genetically distinct but morphologically identical lineages, i.e., assemblages A–H^4,9–11^. Of these, assemblages A and B have a broad host spectrum, infect most vertebrates, and have a high zoonotic risk^12–14^. Assemblages C–H have a high host specificity^4,15^, with assemblages C and D mainly found in canine animals, assemblage E mainly found in hoofed livestock and wildlife, assemblage F found in cats, assemblage G found in rodents, assemblage H found in seals and other marine mammals^9,14^, and assemblages C, D, E, and F identified in human patients^12^. Assemblage E is the most common genotype in cattle globally^4,13,14,16–20^, followed by assemblages A and B^4,13,16^, in addition assemblages C, D^15^, and F have also been reported in cattle^8,15^. In addition, the frequency of infection with zoonotic assemblages A and B from calves was reportedly higher than that with assemblage E, suggesting that calves are associated with a greater risk of transmitting *G. duodenalis* infection to humans^4,21–24^.

The pooled prevalence of bovine *G. duodenalis* detected using molecular methods was ~ 22% globally, and the difference between the highest pooled prevalence (55.4% in Canada) and the lowest pooled prevalence (4.2% in Iran) of giardiasis in different geographic areas is high^14^. Recently, in China, molecular epidemiological surveys of giardiasis in cattle have been conducted in several provinces in recently with differences in prevalence^4,22–48^. Inner Mongolia is located on the northern border of China, spanning 28° 52′ in longitude from east to west with a linear distance of > 2400 km and spanning 15° 59′ in latitude from north to south with a linear distance of 1700 km. Presently, only the prevalence and molecular characterizations of *G. duodenalis* in 108 beef cattle from one farm in the Southwest Alxa Left Banner have been investigated in Inner Mongolia^24^. Hence, this study aimed to investigate the prevalence of *G. duodenalis* in dairy cows in Central Inner Mongolia.

## Methods

### Study areas and sample collection

**Figure 1:**
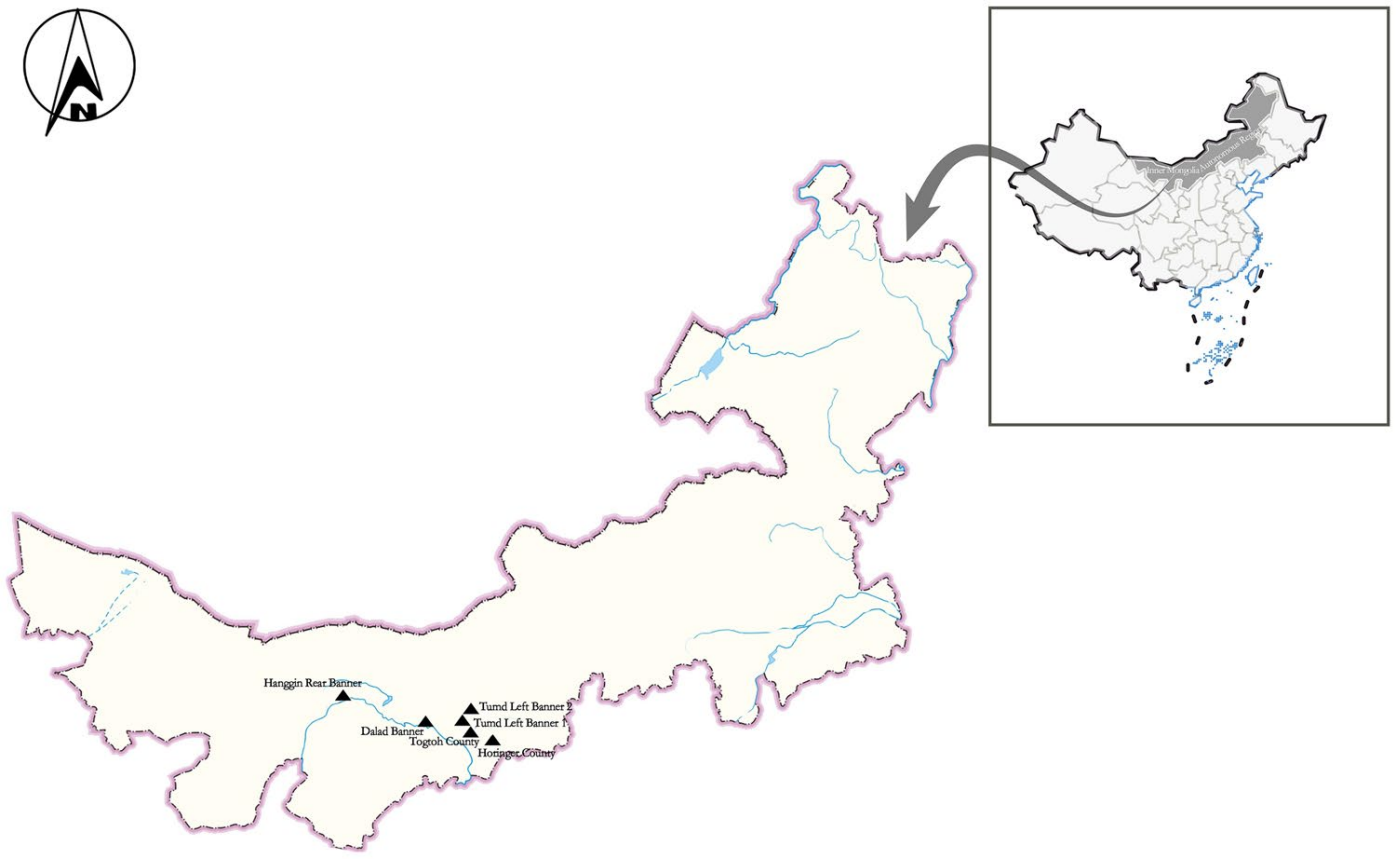
Specific locations from which specimens were collected for this study. Filled triangle: study locations.

From March to September 2021, 505 fresh fecal samples were randomly collected from six dairy farms in the vicinity of Tumd Left Banner, Horinger County, Togtoh County, Dalad Banner, and Hanggin Rear Banner (107° 28′ E–111° 16′ E, 40° 21′ N–40° 35′ N) in Central Inner Mongolia. We have annotated six dairy farms on the map using phptpshop software (Fig. 1). The fecal samples were collected via rectal sampling from dairy cattle or from the inner top layer of fresh feces. The samples included 103 preweaned calves (0–60 days), 105 postweaned calves (61–180 days), 124 young cattle (181–360 days), and 173 adult cattle (> 361 days). Information regarding whether the animals experienced diseases such as diarrhea was recorded during sampling, and the samples were stored at 4 °C before extracting DNA. In the laboratory, the fresh fecal samples was added to a beaker alongside an appropriate amount of distilled water, and then stirred and filtered. The filtrate was centrifuged at 3500×*g* for 10 min and the precipitate was used for DNA extraction.

### DNA extraction and polymerase chain reaction (PCR) amplification

The genomic DNA of 505 fecal samples was extracted E.Z.N. A® Stool DNA Kit (Omega Biotek, Norcross, GA, USA) according to the manufacturer’s protocol and stored at − 20 °C for subsequent experiments. The small subunit ribosomal RNA (SSU rRNA) gene^49^ was amplified via nested PCR (the annealing temperatures for two rounds of PCR were 55 °C and 59 °C) using Premix Taq™ (TaKaRa Taq™ Version 2.0 plus dye) (TaKaRa, Beijing, China) and 1 μL extracted DNA as the template. *SSU rRNA*–positive DNA was subsequently amplified via the nested PCR of *bg*^50^ (the annealing temperatures for two rounds of PCR were 62.7 °C and 55 °C), *gdh*^51^ (the annealing temperatures for two rounds of PCR were 53.7 °C and 56.2 °C), and *tpi*^52^ (the annealing temperatures for two rounds of PCR were 59.5 °C and 56.2 °C). Subsequently, 5 μL PCR products were analyzed via 1.5% agarose gel electrophoresis, and all the PCR products positive for the four genes were sent to a commercial company (Sangon Biotech, Shanghai, China) for sequencing.

### Sequence analysis

The sequences were aligned with reference sequences downloaded from GenBank (http://www.ncbi.nlm.nih.gov) using MEGA 7.0 software (http://www.megasoftware.net/) and the obtained results were analyzed using the BLAST online platform. To comprehensively investigate the relationship among the different isolates, phylogenetic analyses were performed using a concatenated dataset of *bg*, *gdh*, and *tpi* sequences. The specimens successfully subtyped at all the three loci were included in the multilocus genotype (MLG) analysis of *G. duodenalis*, wherein MLG types were identified. Phylogenetic trees were constructed using the neighbor-joining algorithm based on a matrix of evolutionary distances calculated using the Kimura 2-parameter model via MEGA 7.0 software. To assess the robustness of the clusters, 1000 bootstrap replicates were used.

### Statistical analyses

Chi-square test was performed and 95% confidence interval (CI) was obtained using SPSS Statistics 21.0 (IBM Corp., New York, NY, USA) to compare *G. duodenalis* infection rates among the different farms and age groups and diarrhea and nondiarrhea groups. A two-tailed *p-value* of < 0.05 was considered statistically significant.

### Ethics approval and consent to participate

This study was carried out in strict accordance with international standards as published in the “Guide to the feeding, management and use of experimental animals” (8th Edition) and follows the “Regulations on the management of experimental animals” and other relevant laws and regulations. The biomedical research ethics committee of Inner Mongolia Agricultural University specifically approved this study (No. 2020[081]). In addition, permission was obtained from the farm owners before the specimens were collected, and all efforts were made to minimize suffering.

## Results

### *Giardia duodenalis* infection status

Based on the SSU rRNA gene of *G. duodenalis*, 149 positive samples were amplified via PCR of 505 samples, with an overall prevalence of 29.5% (149/505). The overall prevalence of diarrhea and nondiarrhea samples was 31.5% (46/146) and 28.7% (103/359), respectively (Table 1), and the difference was not significant (odds ratio [OR] 0.875; 95% CI 0.576–1.328; p = 0.529).

**Table 1:** Prevalence of *Giardia duodenalis* and assemblages determined via sequence analysis of SSU rRNA.

Farm	Samples size	Age				Total	*p*-value	OR (95% CI)
		Preweaned calves	Postweaned calves	Young cattle	Adult cattle			
Tumd left banner 1	Samples size (diarrhea/nondiarrhea)	20 (8/12)	40 (21/19)	40 (9/31)	40 (17/23)	140 (55/85)	0.006	1.785 (1.181–2.697)
	Positive samples size (Diarrhea/Nondiarrhea)	3 (0/3)	22 (9/13)	13 (2/11)	16 (7/9)	54 (18/36)		
	Overall prevalence (prevalence of diarrhea/nondiarrhea samples) (%)	15% (0/25%)	55% (42.9%/68.4%)	32.5% (22.2%/35.5%)	40% (41.2%/39.1%)	38.6% (32.7%/42.4%)		
	SSU rRNA (n)	E (3)	E (17), A (5)	E (13)	E (16)	E (49), A (5)		
Tumd left banner 2	Samples size (Diarrhea/Nondiarrhea)	30 (2/28)	20 (9/11)	20 (1/19)	40 (0/40)	110 (12/98)	0.197	0.728 (0.449–1.181)
	Positive samples size (Diarrhea/Nondiarrhea)	8 (0/8)	8 (3/5)	5 (1/4)	6 (0/6)	27 (4/23)		
	Overall prevalence (Prevalence of diarrhea/nondiarrhea samples) (%)	26.7% (0/28.6%)	40% (33.3%/45.5%)	25% (100%/21.1%)	15% (0/15%)	24.5% (33.3%/23.5%)		
	*SSU* rRNA (n)	E (8)	E (7)	E (5)	E (5), A (1)	E (25), A (1)		
Horinger county	Samples size (Diarrhea/Nondiarrhea)	23 (8/15)	20 (18/2)	41 (7/34)	36 (7/29)	120 (40/80)	0.031	0.589 (0.363–0.956)
	Positive samples size (Diarrhea/Nondiarrhea)	0 (0/0)	9 (7/2)	9 (2/7)	8 (2/6)	26 (11/15)		
	Overall prevalence (Prevalence of diarrhea/nondiarrhea samples) (%)	0 (0/0)	45% (38.9%/100%)	22.0% (28.6%/20.6%)	22.2% (28.6%/20.7%)	21.7% (27.5%/18.8%)		
	SSU rRNA (n)	–	E (8), A (1)	E (8), A (1)	E (7), A (1)	E (23), A (3)		
Togtoh county	Samples size (diarrhea/nondiarrhea)	30 (12/18)	20 (20/0)	20 (3/17)	40 (4/36)	110 (39/71)	0.548	1.15 0.729–1.816)
	Positive samples size (Diarrhea/Nondiarrhea)	0 (0/0)	11 (11/0)	13 (1/12)	11 (1/10)	35 (13/22)		
	Overall prevalence (prevalence of diarrhea/nondiarrhea samples) (%)	0 (0/0)	55% (55%/0)	65% (33.3%/70.6%)	27.5% (25%/27.8%)	31.8% (33.3%/31.0%)		
	SSU rRNA (n)	–	E (11)	E (9), A (2)	E (10), A (1)	E (30), A (3)		
Dalad banner	Samples size (diarrhea/nondiarrhea)	0 (0/0)	5 (0/5)	3 (0/3)	9 (0/9)	17 (0/17)	0.594	1.316 (0.478–3.626)
	Positive samples size (Diarrhea/Nondiarrhea)	0 (0/0)	0 (0/0)	3 (0/3)	3 (0/3)	6 (0/6)		
	Overall prevalence (prevalence of diarrhea/nondiarrhea samples) (%)	0 (0/0)	0 (0/0)	100% (0/100%)	33.3% (0/33.3%)	35.3% (0/35.3%)		
	SSU rRNA (n)	–	–	E (2), A (1)	E (3)	E (5), A (1)		
Hanggin rear Banner	Samples size (diarrhea/nondiarrhea)	0 (0/0)	0 (0/0)	0 (0/0)	8 (0/8)	8 (0/8)	0.288	0.337 (0.041–2.762)
	Positive samples size (Diarrhea/Nondiarrhea)	0 (0/0)	0 (0/0)	0 (0/0)	1 (0/1)	1 (0/1)		
	Overall prevalence (prevalence of diarrhea/nondiarrhea samples) (%)	0 (0/0)	0 (0/0)	0 (0/0)	12.5% (0/12.5%)	12.5% (0/12.5%)		
	SSU rRNA (n)	–	–	–	E (1)	E (1)		
Total	Samples size (diarrhea/nondiarrhea)	103 (30/73)	105 (68/37)	124 (20/104)	173 (28/145)	505 (146/359)	–	–
	Positive samples size (Diarrhea/Nondiarrhea)	11 (0/11)	50 (30/20)	43 (6/37)	45 (10/35)	149 (46/103)		
	Overall prevalence (prevalence of diarrhea/nondiarrhea samples) (%)	10.7% (0/15.1%)	47.6% (44.1%/54.1%)	34.7% (30%/35.6%)	26.0% (35.7%/24.1%)	29.5% (31.5%/28.7%)		
	SSU rRNA (n)	E (11)	E (43), A (6)	E (37), A (4)	E (42), A (3)	E (133), A (13)		
*p*-value	–	< 0.001	< 0.001	0.146	0.214	–	–	–
OR (95% CI)	–	0.229 (0.118–0.442)	2.764 (1.771–4.314)	1.377 (0.894–2.122)	0.771 (0.511–1.163)			

The dash (–) indicates that no data were obtained.

The overall prevalence of *G. duodenali* in the six sampling farms was 38.6% (54/140), 24.5% (27/110), 21.7% (26/120), 31.8% (35/110), 35.3% (6/17), and 12.5% (1/8). The difference in the Tumd Left Banner 1 field was highly significant compared with that in the other fields (OR 1.785; 95% CI 1.181–2.697; p = 0.006); Horinger County field had also showed a significant difference (OR 0.589; 95% CI 0.363–0.956; p = 0.031). The prevalence *G. duodenalis* in the diarrhea samples from the six sampling sites was 32.7% (18/55), 33.3% (4/12), 27.5% (11/40), 33.3% (13/39), 0 (0/0), and 0 (0/0) (Table 1), and the difference in prevalence among the sampling sites with diarrhea was not significant (p > 0.05).

The overall prevalence of *G. duodenalis* in all the samples in the four age groups was 10.7% (11/103), 47.6% (50/105), 34.7% (43/124), and 26.0% (45/173). The difference in preweaned calves was highly significant compared with that in the other age groups (OR 0.229; 95% CI 0.118–0.442; p < 0.001); the difference in postweaned calves was highly significant (OR 2.764; 95% CI 1.771–4.314; p < 0.001). The prevalence of *G. duodenalis* in the diarrhea samples of the four age groups were 0 (0/30), 44.1% (30/68), 30% (6/20), and 35.7% (10/28) (Table 1), with a highly significant difference in postweaned calves compared with the other age groups (OR 3.059; 95% CI 1.476–6.341; p = 0.002).

In total, 149 positive samples were used for the PCR amplification of *SSU rRNA*; however, sequence analyses revealed that 146 of them were plausible sequences, with 13 (8.9%, 13/146) for *G. duodenalis* assemblages A and 133 (91.1%, 133/146) for assemblage E. Assemblage E was detected in all the four age groups. Only assemblage E was present in Hanggin Rear Banner field and in some age groups in other fields. Assemblage A was not observed in preweaned calves at any of the sites (Table 1).

### *Giardia* species identification and analysis

In the 149 positive samples, 38, 47, and 70 plausible sequences were obtained via PCR amplification and gene sequencing of *gdh*, *tpi*, and *bg* of *G. duodenalis*, respectively. Of the 38 isolates of *gdh* (Table 2), one isolate was identified as 1 assemblage A sequence (A1) and 37 were identified as 12 assemblage E sequences, including E1 (n = 17), E2 (n = 9), E3 (n = 1), E4 (n = 1), E5 (n = 2), and one each for E6–E12. Furthermore, of the 47 isolates of *tpi* (Table 2), three isolates were identified as 1 assemblage A sequence (A1) and 44 were identified as 23 assemblage E sequences, including E1 (n = 11), E2 (n = 6), E3 (n = 1), E4 (n = 2), E5 (n = 3), E19 (n = 2), E20 (n = 3), E6–E18 (n = 1), and one each for E21–E23. Of the 70 isolates of *bg* (Table 2), two isolates were identified as 1 assemblage A sequence (A1) and 68 were identified as 24 assemblage E sequences, including E1 (n = 22), E2 (n = 4), E3 (n = 7), E4 (n = 9), E5 (n = 3), E6 (n = 2), E7 (n = 3), E8 (n = 1), E9 (n = 2), and one each for E10–E24. The sequence alignment analysis of the above four genes identified differences in the genotypes of seven samples alongside mixed infections (assemblage A + E) (Table 3).

**Table 2:** Intra-assemblage substitutions and insertion in *gdh*, *tpi*, and *bg* sequences within *Giardia duodenalis* assemblage E.

Subtype (no.) *gdh*	Nucleotide positions																									
	105	133	141	282	303	318	336	378	435	444	453	455	457	461	468	469	471	472	473	481	483	485	498			
Ref. sequence (GenBank ID: MK645797)	C	A	A	T	C	G	A	G	G	C	T	G	G	T	T	G	A	G	G	C	T	G	G			
E1 (17)	–	–	–	–	–	–	–	–	–	–	–	–	–	–	–	–	–	–	–	–	–	–	–			
E2 (9)	T	–	–	–	–	–	–	–	–	–	–	–	–	–	–	–	–	–	–	–	–	–	–			
E3 (1)	–	–	–	–	–	–	–	–	–	–	–	–	–	–	–	–	–	–	A	–	–	–	A			
E4 (1)	T	–	–	G	–	–	–	–	–	–	–	–	–	–	–	–	–	–	–	–	–	–	–			
E5 (2)	–	–	G	–	–	–	–	–	–	–	–	–	–	–	–	–	–	–	–	–	–	–	–			
E6 (1)	–	–	–	–	–	–	–	–	–	–	–	A	–	–	–	–	–	–	–	–	–	–	–			
E7 (1)	–	–	–	–	T	A	–	–	–	–	–	–	–	–	–	–	–	–	–	–	–	–	–			
E8 (1)	–	–	–	–	–	–	–	–	–	–	–	–	–	–	–	–	–	–	A	–	–	–	–			
E9 (1)	–	–	–	–	–	–	–	C	C	G	G	–	C	A	C	C	C	T	–	T	C	T	–			
E10 (1)	T	C	–	–	–	–	–	–	–	–	–	–	–	–	–	–	–	–	–	–	–	–	–			
E11 (1)	T	–	–	–	–	–	–	–	–	–	–	A	–	–	–	–	–	–	–	–	–	–	–			
E12 (1)	–	–	G	G	–	–	G	–	–	–	–	–	–	–	–	–	–	–	–	–	–	–	–			

*Tpi*	50	71	83	87	88	119	124	138	145	186	243	264	285	302	312	335	338	342	377	381	399	450	455	468		
Ref. sequence (GenBank ID: MH079444)	T	T	T	G	*	A	T	A	A	A	C	G	C	A	C	A	A	T	A	G	C	A	A	A		
E1 (11)	–	–	–	–	–	–	–	–	–	–	–	–	–	–	–	–	–	–	–	–	–	–	–	–		
E2 (6)	–	C	–	–	–	–	–	–	–	–	–	–	–	–	–	–	–	–	–	–	–	–	–	–		
E3 (1)	–	C	–	–	–	–	–	G	–	–	–	–	–	–	–	–	–	–	–	–	–	–	–	–		
E4 (2)	–	C	–	–	–	–	–	–	–	–	T	–	–	–	–	–	–	–	–	–	–	–	–	–		
E5 (3)	C	C	G	A	–	–	–	–	–	–	–	–	–	–	–	–	–	–	–	–	–	–	–	–		
E6 (1)	–	C		–	–	–	–	–	–	–	–	–	–	–	T	–	G	–	–	–	–	–	–	–		
E7 (1)	–	–	–	–	–	–	–	–	–	–	–	–	–	–	–	–	–	–	–	–	T	–	–	–		
E8 (1)	–	–	–	–	–	–	–	–	–	–	–	–	T	–	–	–	–	–	–	–	–	–	–	–		
E9 (1)	–	–	–	–	–	–	–	–	–	G	–	–	–	–	–	–	–	–	–	–	–	–	–	–		
E10 (1)	–	–	–	–	–	G	–	–	–	–	–	–	–	–	–	–	–	–	–	A	–	–	–	–		
E11 (1)	–	C	–	–	–	–	–	–	–	–	–	–	–	T	–	–	–	–	–	–	–	–	–	–		
E12 (1)	C	C	–	–	–	–	–	–	–	–	T	–	–	–	–	–	–	–	–	–	–	–	–	–		
E13 (1)	–	C	–	–	–	–	–	–	G	–	T	–	–	–	–	–	–	–	G	–	–	G	–	G		
E14 (1)	–	C	–	A	–	–	–	–	–	–	T	–	–	–	–	–	–	–	–	–	–	G	C	G		
E15 (1)	–	C	–	A	–	–	–	–	–	–	T	–	–	–	–	–	–	–	–	–	–	–	–	–		
E16 (1)	–	C	G	A	–	–	–	–	–	–	–	–	–	–	–	–	–	–	–	–	–	–	C	–		
E17 (1)	C	C	G	A	G	–	–	–	–	–	T	–	–	–	–	–	–	–	–	–	–	–	–	–		
E18 (1)	C	C	–	–	–	–	C	–	–	–	–	–	–	–	T	–	G	–	–	–	–	–	–	–		
E19 (1)	–	–	–	–	–	–	–	G	–	–	–	A	–	–	–	–	–	–	–	–	–	–	–	–		
E20 (2)	–	C	–	A	–	–	–	–	–	–	–	–	–	–	–	–	–	–	–	–	–	–	–	–		
E21 (3)	C	C	–	A	–	–	–	–	–	–	–	–	–	–	–	G	–	–	–	–	–	–	–	–		
E22 (1)	C	C	–	A	–	–	–	–	–	–	–	–	–	–	–	–	–	–	–	–	–	–	–	–		
E23 (1)	–	C	–	A	–	–	–	–	–	–	–	–	–	–	–	–	–	G	–	–	–	–	–	–		

*Bg*	68	69	77	103	104	107	124	129	155	156	175	203	232	259	277	304	316	319	348	368	418	431	436	439	457	463
Ref. sequence (GenBank ID: MK610389)	T	*	*	G	A	A	C	A	G	C	A	G	G	A	C	C	G	C	G	G	C	G	C	C	T	G
E1 (22)	–	–	–	–	–	–	–	–	–	–	–	–	–	–	–	–	–	–	–	–	–	–	–	–	–	–
E2 (4)	–	–	–	–	–	–	–	–	–	–	–	–	–	–	–	–	–	–	–	–	T	–	–	–	–	–
E3 (7)	–	–	–	–	–	–	–	–	–	–	–	–	–	–	T	–	–	–	–	–	T	–	–	–	–	–
E4 (9)	C	–	–	–	–	–	–	–	–	–	–	–	–	–	–	–	–	–	–	–	T	–	–	–	–	–
E5 (3)	C	–	–	–	–	–	–	–	–	–	–	–	–	–	–	–	–	–	–	–	–	–	–	–	–	–
E6 (2)	–	–	–	–	–	–	–	–	–	–	G	–	–	–	–	–	–	–	–	–	–	–	–	–	–	–
E7 (3)	C	–	–	–	–	–	–	–	–	–	G	–	–	G	–	–	–	–	–	–	–	–	–	–	–	–
E8 (1)	C	–	–	–	–	–	–	–	–	–	–	–	–	–	–	T	–	–	–	–	–	–	–	–	–	–
E9 (2)	C	–	–	–	–	–	–	–	–	–	–	–	–	–	–	T	–	–	–	–	T	–	–	–	–	–
E10 (1)	–	–	–	–	–	–	–	–	–	–	G	–	–	–	–	–	–	–	–	–	–	–	T	–	–	–
E11 (1)	–	–	–	–	–	–	–	–	A	–	–	–	–	–	–	–	–	–	–	–	–	–	–	–	–	–
E12 (1)	–	–	–	–	–	–	–	–	–	–	–	A	–	–	–	–	–	–	–	–	–	–	–	–	–	–
E13 (1)	–	–	–	–	–	–	–	–	–	–	–	–	–	–	T	–	–	–	–	–	–	–	–	–	–	–
E14 (1)	C	T	–	–	–	–	–	–	–	–	–	–	–	–	–	–	–	–	–	–	–	–	–	–	–	–
E15 (1)	–	–	A	–	–	–	–	–	–	–	–	–	–	–	–	–	–	–	–	–	T	–	–	–	–	–
E16 (1)	C	–	–	–	T	G	–	G	–	–	–	–	A	–	–	–	–	–	–	–	T	–	–	–	–	–
E17 (1)	–	–	–	–	–	–	–	–	–	–	–	–	–	–	–	–	–	–	–	–	T	–	–	–	–	A
E18 (1)	–	–	–	–	–	–	–	–	–	T	G	–	–	–	–	–	–	–	–	–	–	A	–	T	–	–
E19 (1)	–	–	–	A	–	–	–	–	–	–	G	–	–	–	–	–	–	–	–	A	–	–	–	–	–	–
E20 (1)	–	–	–	–	–	–	–	–	–	–	G	–	–	–	–	–	–	–	A	–	–	–	–	T	–	–
E21 (1)	–	–	–	–	–	–	T	–	–	–	G	–	–	–	–	–	–	–	–	–	–	–	–	–	–	–
E22 (1)	–	–	–	–	–	–	–	–	–	–	G	–	–	–	T	–	–	–	–	–	T	–	–	–	–	–
E23 (1)	–	–	–	–	–	–	–	–	–	–	–	–	–	–	–	–	A	T	–	–	T	–	–	–	C	–
E24 (1)	–	–	–	–	–	–	–	–	–	–	G	–	–	G	–	–	–	–	–	–	–		–	–	–	–

The dash (–) indicates that the sequence is the same as the reference sequence. The asterisk (*) indicates the insertion site of the reference sequence.

**Table 3:** Multilocus characterization of *Giardia duodenalis* isolates based on *bg*, *gdh*, and *tpi* sequences.

Farm	Age	Cattle ID	SSU rRNA (GenBank accession no.)	*gdh* (GenBank accession no.)	*tpi* (GenBank accession no.)	*bg* (GenBank accession no.)	MLG type	Mix
Tumd left banner 1	Preweaned calves	T1–2	OP189375 (E)		OP189521 (E1)	OP189568 (E1)		
		T1–4	OP189376 (E)					
		T1–15	OP189377 (E)					
	Postweaned calves	T1–22	OP189378 (A)					
		T1–23	OP189379 (E)			OP189606 (E2)		
		T1–26	OP189380 (E)		OP189550 (A1)	OP189569 (E1)		A + E
		T1–28	OP189381 (E)					
		T1–30	OP189382 (A)	OP189640 (A1)		OP189570 (E1)		A + E
		T1–32	OP189383 (E)					
		T1–36	OP189384 (A)					
		T1–38	OP189385 (E)	OP189641 (E1)				
		T1–39	OP189476 (A)		OP189551 (E1)			A + E
		T1–44	OP189386 (E)					
		T1–46	OP189387 (E)			OP189611 (E23)		
		T1–47	OP189388 (E)	OP189649 (E2)		OP189571 (E4)		
		T1–48	OP189389 (E)			OP189605 (E4)		
		T1–49	OP189390 (E)					
		T1–51	OP189391 (E)	OP189642 (E12)		OP189631 (E1)		
		T1–52	OP189476 (E)					
		T1–53	OP189392 (E)	OP189670 (E1)				
		T1–54	OP189393 (A)		OP189522 (A1)	OP189572 (A1)		
		T1–56	OP189394 (E)			OP189607 (E4)		
		T1–57	OP189395 (E)					
		T1–58	OP189396 (E)		OP189552 (E1)			
		T1–60	OP189397 (E)	OP189650 (E2)	OP189553 (E23)	OP189573 (E1)	MLG E12	
	Young cattle	T1–105	OP189480 (E)					
		T1–110	OP189410 (E)	OP189643 (E10)	OP189554 (E1)	OP189576 (E8)	MLG E22	
		T1–111	OP189481 (E)					
		T1–112	OP189411 (E)			OP189633 (E3)		
		T1–113	OP189482 (E)	OP189651 (E6)		OP189614 (E4)		
		T1–120	OP189483 (E)					
		T1–122	OP189484 (E)					
		T1–125	OP189485 (E)					
		T1–130	OP189412 (E)		OP189555 (E1)	OP189615 (E1)		
		T1–131	OP189413 (E)		OP189556 (E2)			
		T1–133	OP189414 (E)					
		T1–139	OP189486 (E)		OP189566 (E5)	OP189634 (E1)		
		T1–140	OP189487 (E)					
	Adult cattle	T1–63	OP189515 (E)			OP189632 (E1)		
		T1–64	OP189398 (E)					
		T1–68	OP189399 (E)					
		T1–69	OP189400 (E)		OP189523 (E2)			
		T1–70	OP189401 (E)					
		T1–76	OP189402 (E)					
		T1–78	OP189478 (E)					
		T1–79	OP189403 (E)			OP189612 (E1)		
		T1–80	OP189404 (E)					
		T1–82	OP189405 (E)					
		T1–84	OP189479 (E)					
		T1–90	OP189406 (E)			OP189574 (E11)		
		T1–96	OP189407 (E)					
		T1–97	OP189516 (E)					
		T1–98	OP189408 (E)			OP189575 (E12)		
		T1–99	OP189409 (E)		OP189524 (E2)	OP189608 (E19)		
Total	–	–	E (49), A (5)	E (7), A (1)	E (10), A (2)	E (20), A (1)		
Tumd left banner 2	Preweaned calves	T2–1	OP189417 (E)	OP189664 (E1)	OP189525 (E5)	OP189577 (E9)	MLG E1	
		T2–2	OP189418 (E)					
		T2–6	OP189419 (E)			OP189609 (E3)		
		T2–12	OP189489 (E)					
		T2–21	OP189420 (E)					
		T2–22	OP189421 (E)	OP189665 (E1)	OP189526 (E19)	OP189578 (E1)	MLG E2	
		T2–25	OP189422 (E)					
		T2–29	OP189423 (E)	OP189666 (E2)	OP189527 (E19)	OP189579 (E5)	MLG E13	
	Postweaned calves	T2–31	OP189424 (E)	OP189644 (E5)	OP189561 (E20)	OP189610 (E1)	MLG E21	
		T2–38	OP189425 (E)			OP189580 (E1)		
		T2–42	OP189426 (E)	OP189667 (E3)	OP189562 (E2)	OP189581 (E4)	MLG E19	
		T2–43	OP189427 (E)	OP189668 (E2)	OP189563 (E1)	OP189603 (E2)	MLG E14	
		T2–45	OP189428 (E)		OP189529 (E21)			
		T2–46	OP189429 (E)	OP189672 (E5)	OP189528 (E2)			
		T2–49	OP189430 (E)			OP189582 (E9)		
	Young cattle	T2–54	OP189431 (E)					
		T2–57	OP189432 (E)					
		T2–63	OP189433 (E)					
		T2–65	OP189490 (E)					
		T2–68	OP189434 (E)	OP189669 (E1)	OP189530 (E16)	OP189583 (E4)	MLG E8	
	Adult cattle	T2–73	OP189491 (E)			OP189613 (E15)		
		T2–85	OP189492 (E)		OP189531 (E3)	OP189604 (E5)		
		T2–97	OP189435 (A)					
		T2–99	OP189488 (E)		OP189532 (E13)	OP189584 (E18)		
		T2–103	OP189415 (E)	OP189674 (E8)	OP189533 (E22)	OP189616 (E20)	MLG E23	
		T2–105	OP189416 (E)	OP189638 (E1)		OP189635 (E1)		
Total	–	–	E (25), A (1)	E (10)	E (12)	E (15)		
Horinger county	Preweaned calves	0						
	Postweaned calves	He-101	OP189461 (A)					
		He-103	OP189462 (E)	OP189648 (E11)	OP189567 (E10)	OP189596 (E3)	MLG E25	
		He-104	OP189463 (E)		OP189547 (E15)	OP189597 (E1)		
		He-107	OP189464 (E)					
		He-108	OP189465 (E)			OP189598 (E1)		
		He-112	OP189466 (E)	OP189671 (E4)	OP189548 (E2)	OP189599 (E6)	MLG E20	
		He-117	OP189467 (E)					
		He-118	OP189468 (E)					
		He-120	OP189469 (E)	OP189675 (E1)	OP189549 (E1)	OP189630 (E10)	MLG E9	
	Young cattle	He-62	OP189455 (E)					
		He-63	OP189456 (E)			OP189595 (E3)		
		He-65	OP189512 (E)	OP189647 (E7)	OP189545 (E18)	OP189626 (E17)	MLG E24	
		He-66	OP189457 (E)					
		He-78	OP189458 (E)					
		He-89	OP189459 (E)		OP189546 (E11)			
		He-92	OP189513 (E)			OP189629 (E2)		
		He-93	OP189460 (A)					
		He-96	OP189514 (E)					
	Adult cattle	He-26	OP189448 (E)			OP189593 (E1)		
		He-29	OP189449 (E)					
		He-32	OP189450 (E)					
		He-35	OP189451 (E)			OP189627 (E22)		
		He-43	OP189452 (E)			OP189625 (E6)		
		He-54	OP189453 (E)					
		He-55	OP189511 (E)			OP189594 (E1)		
		He-56	OP189454 (A)			OP189628 (E1)		A + E
Total	–	–	E (23), A (3)	E (4)	E (6)	E (13)		
Togtoh county	Preweaned calves	0						
	Postweaned calves	Tuo-31	OP189436 (E)	OP189652 (E1)	OP189534 (E17)	OP189585 (E7)	MLG E3	
		Tuo-32	OP189437 (E)	OP189653 (E1)	OP189535 (E4)	OP189586 (E7)	MLG E6	
		Tuo-33	OP189438 (E)	OP189654 (E2)	OP189536 (E1)	OP189587 (E13)	MLG E16	
		Tuo-34	OP189439 (E)	OP189655 (E1)	OP189537 (E5)	OP189588 (E14)	MLG E10	
		Tuo-36	OP189440 (E)	OP189656 (E1)	OP189538 (E12)	OP189589 (E5)	MLG E4	
		Tuo-39	OP189493 (E)	OP189657 (E2)	OP189539 (E6)	OP189590 (E3)	MLG E17	
		Tuo-40	OP189494 (E)	OP189639 (E1)	OP189564 (E1)	OP189636 (E3)	MLG E11	
		Tuo-41	OP189441 (E)	OP189658 (E1)	OP189540 (E20)	OP189591 (E1)	MLG E5	
		Tuo-42	OP189442 (E)	OP189659 (E1)	OP189541 (E4)	OP189592 (E7)	MLG E6	
		Tuo-44	OP189443 (E)	OP189660 (E1)	OP189565 (E9)			
		Tuo-45	OP189444 (E)	OP189661 (E1)	OP189557 (E7)	OP189617 (E24)	MLG E7	
	Young cattle	Tuo-71	OP189499 (A)	OP189662 (E2)	OP189558 (A1)			A + E
		Tuo-73	OP189500 (A)		OP189559 (E8)	OP189623 (A1)		A + E
		Tuo-74	OP189501 (E)					
		Tuo-75	OP189447 (E)	OP189645 (E2)	OP189542 (E14)	OP189600 (E2)	MLG E15	
		Tuo-76	OP189502 (E)					
		Tuo-84	OP189503 (E)	OP189663 (E2)	OP189543 (E1)	OP189601 (E1)	MLG E18	
		Tuo-85	OP189518 (E)			OP189619 (E4)		
		Tuo-87	OP189519 (E)		OP189544 (E1)	OP189602 (E1)		
		Tuo-88	OP189504 (E)					
		Tuo-89	OP189505 (E)			OP189637 (E3)		
		Tuo-90	OP189520 (E)		OP189560 (E20)			
	Adult cattle	Tuo-51	OP189445 (E)			OP189618 (E21)		
		Tuo-53	OP189495 (E)					
		Tuo-54	OP189496 (E)					
		Tuo-57	OP189497 (E)					
		Tuo-60	OP189498 (A)			OP189622 (E16)		A + E
		Tuo-68	OP189517 (E)	OP189673 (E9)				
		Tuo-70	OP189446 (E)					
		Tuo-92	OP189506 (E)	OP189646 (E1)		OP189620 (E1)		
		Tuo-93	OP189507 (E)					
		Tuo-94	OP189508 (E)					
		Tuo-95	OP189509 (E)					
Total	–	–	E (30), A (3)	E (16)	E (16), A (1)	E (18), A (1)		
Dalad banner	Preweaned calves	0						
	Postweaned calves	0						
	Young cattle	Da-6	OP189470 (A)					
		Da-7	OP189471 (E)			OP189621 (E4)		
		Da-8	OP189472 (E)			OP189624 (E4)		
	Adult cattle	Da-11	OP189473 (E)					
		Da-15	OP189474 (E)					
		Da-17	OP189475 (E)					
Total	–	–	E (5), A (1)	0	0	E (2)		
Hanggin rear banner	Preweaned calves	0						
	Postweaned calves	0						
	Young cattle	0						
	Adult cattle	Ba-4	OP189510 (E)					
Total	–	–	E (1)	0	0	0		
Total		–	E (133), A (13)	E (37), A (1)	E (44), A (3)	E (68), A (2)		

**Figure 2:**
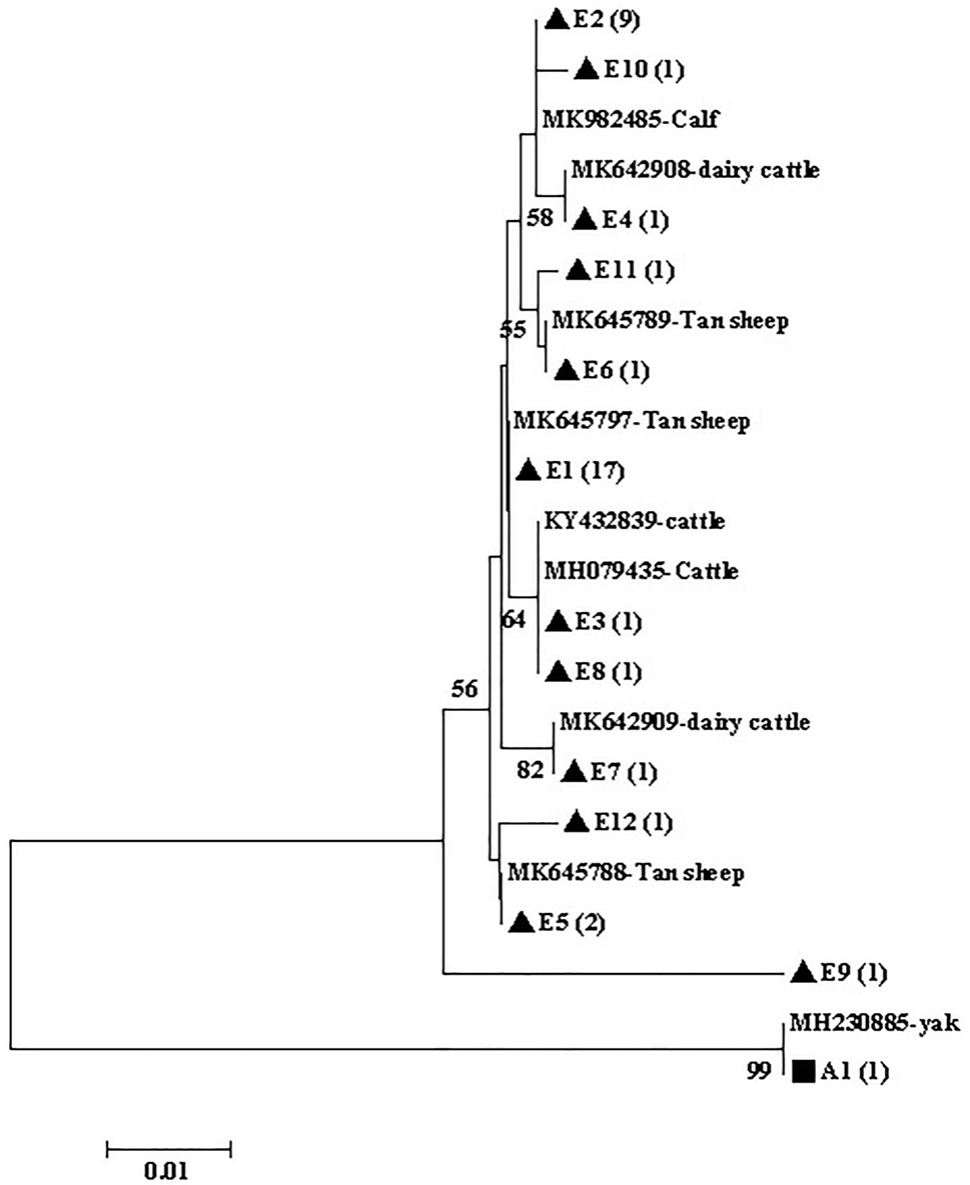
Phylogenetic tree of *Giardia duodenalis* based on *gdh* sequences. The phylogenetic tree was inferred via neighbor-joining analysis of genetic distances calculated using the Kimura 2-parameter model. Percent bootstrap values of > 50% from 1000 replicates are shown to the left of nodes. The isolates indicated in black triangles (filled triangle) and black squares (filled square) represent assemblages E and A, respectively, identified in cattle in this study.

**Figure 3:**
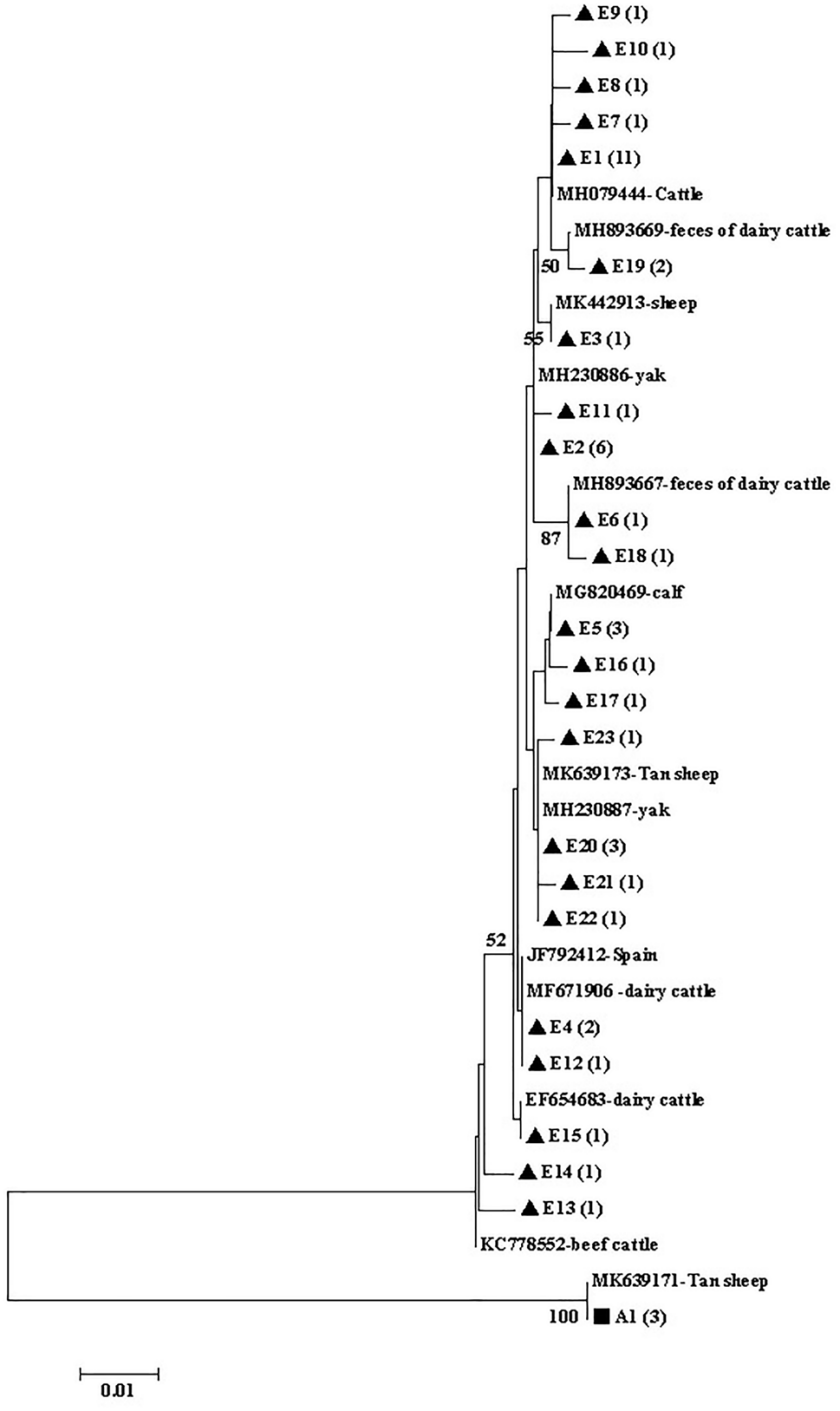
Phylogenetic tree of *Giardia duodenalis* based on *tpi* sequences. The phylogenetic tree was inferred via neighbor-joining analysis of genetic distances calculated using the Kimura 2-parameter model. Percent bootstrap values > 50% from 1000 replicates are shown to the left of nodes. Assemblages E and A isolates identified in cattle in this study are indicated in black triangles (filled triangle) and black squares (filled square), respectively.

**Figure 4:**
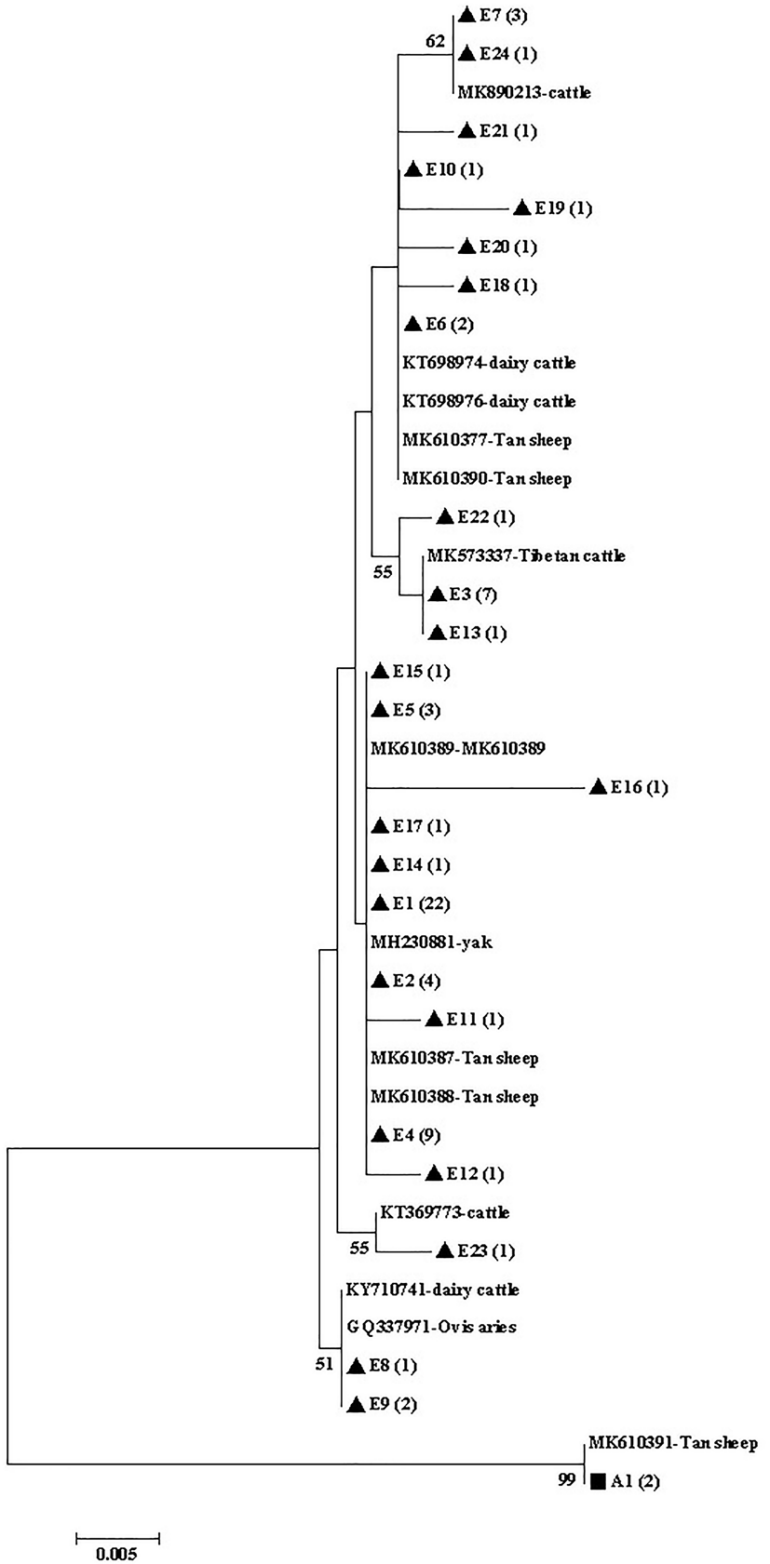
Phylogenetic tree of *Giardia duodenalis* based on *bg* sequences. The phylogenetic tree was inferred via the neighbor-joining analysis of genetic distances calculated using the Kimura 2-parameter model. Percent bootstrap values > 50% from 1000 replicates are shown to the left of nodes. The black triangles (filled triangle) and black squares (filled square) represent assemblage E and assemblage A, respectively, identified in cattle in this study.

The phylogenetic analysis of *gdh* (Fig. 2), *tpi* (Fig. 3), and *bg* (Fig. 4) sequences based on *G. duodenalis* revealed that the phylogenetic tree of the three genes was divided into two branches (assemblages A and E).

### Assemblage E multilocus genotype (MLG) distribution

**Figure 5:**
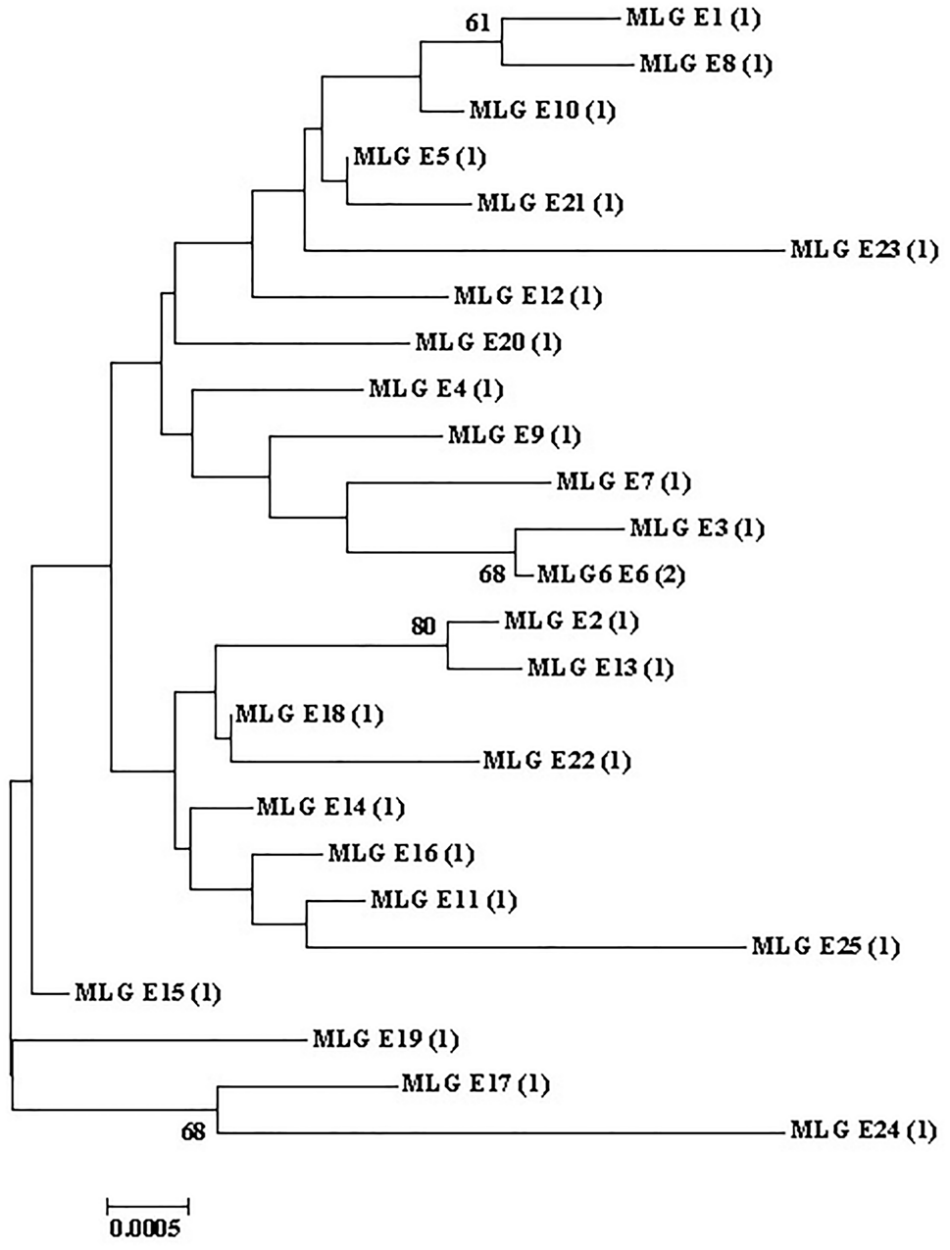
Phylogenetic relationships between *Giardia duodenalis* assemblage E MLGs. The phylogenetic tree was constructed using a concatenated dataset of *bg*, *tpi*, and *gdh* sequences, and the neighbor-joining analysis revealed identical topologies.

The sequences of *gdh*, *tpi*, and *bg* were successfully obtained from 26 isolates, and three genes from 26 isolates were combined for genotyping, forming 25 different assemblage E multilocus genotypes (MLGs) (Table 3). Phylogenetic analysis revealed that all assemblage E MLGs formed six subgroups (Fig. 5).

## Discussion

*Giardia duodenalis* is an important intestinal parasite. To date, numerous studies worldwide have reported *G. duodenalis* infection in cattle^14^. Bovine giardiasis is also widespread in China^4,13,14,24–48^. Herein, a molecular epidemiological investigation of *G*. *duodenalis* was conducted involving 505 dairy cattle fecal samples from six sites in Central Inner Mongolia. The findings filled the gaps in the data regarding *G. duodenalis* infection in dairy cattle in Inner Mongolia as well as reconfirmed the existence of *G. duodenalis* infection in animals in Inner Mongolia^44,53–55^. The overall prevalence of *G. duodenalis was* 29.5% (149/505) in this study, which exceeded the global pooled prevalence of 22%^14^ and was higher than the pooled prevalence of 14.1% in Chinese cattle^14^. In China, the prevalence of *G. duodenalis* in this study was only lower than the prevalence of *G*. *duodenalis* in cattle in Shanghai (60.1%, 492/818)^32^, Sichuan (41.2%, 126/306)^34^, and Guangdong (74.2%, 288/388)^38^ and higher than the prevalence data reported in Xinjiang (13.4%, 69/514)^4^; Ningxia (4.38%, 74/1688)^37^ and (2.12%, 29/1366)^24^; Heilongjiang (5.2%, 42/814)^25,26^, (4.98%, 16/321)^27^ and (15.4%, 8/52)^28^; Jilin (6.63%, 25/377)^28^; Liaoning (8.4%, 19/226)^28^; Hubei ( 22.7%, 77/339)^29^; Shandong (13.04%, 9/69, PCR) and (18.84%, 13/69, rapid kit)^30^; Shaanxi (18.87%, 70/371)^31^; Sichuan (9.4%, 26/278)^33^; Beijing (1.70%, 14/822)^35^; Gansu (1.0%, 14/1414)^36^ and (2.63%, 33/1257)^37^; Guangdong (2.2%, 31/1440)^39^; Hebei and Tianjin (4.7%, 49/1040)^40^; Henan (7.2%, 128/1777)^41^; Jiangsu (20.6%, 281/1366)^42^; Jiangxi (9.2%, 52/566)^43^; Qinghai (10%, 39/389)^45^; Tibet (3.8%, 17/442)^46^; Yunnan (10.49%, 41/ 391)^47^; Taiwan (19.87%, 31/156)^48^ and that reported in the only survey on *G. duodenalis* in Inner Mongolia (9.3%, 10/108)^44^. However, it is difficult to compare prevalence data because they are influenced by a range of factors, including study design, diagnostic method, geographical conditions, total number of samples, age of animals, and sampling season^4^. The high prevalence of this study may also be related to the high density of local cattle farming. In addition, there were significant differences in the overall prevalence of *G. duodenalis* among the sampling sites in this study. Furthermore, positive samples were detected in all the age groups, indicating that all the age groups of cows are susceptible to *G. duodenalis*^16,38^.

Herein, neither the overall prevalence nor the prevalence in the different sampling sites of *G. duodenalis* was correlated with the presence of diarrhea in the sampled animals. Additionally, *G. du*odenalis was not detected in the diarrhea samples of preweaned calves. This observation is inconsistent with the combined global data, as the latter reported a significant correlation between *G. duodenalis* infection and cattle having diarrhea and preweaned calves^14^. The causes for diarrhea in animals are complex, with the pathogens including various viruses, bacteria, and parasites^56^. In addition, in *G. duodenalis* infection, the appearance of symptoms is also related to the stage of the infection. However, in the diarrhea samples in the present study, the prevalence of *G. duodenalis* in postweaned calves was significantly higher than that in the other age groups. In addition, the overall prevalence in the preweaned calves was significantly lower than that in the other age groups and that in postweaned calves was significantly higher than that in the other age groups in all the samples, which does not reflect the decrease in *G. duodenalis* infection rates with increasing age as reported in the literature^42^. The high prevalence of *G. duodenalis* in postweaned calves has also been reported in China^4,27,29^, Norway^57^, Germany^58^, USA^59,60^, and Canada^61^. However, there are also several studies reporting a relatively high prevalence of *G. duodenalis* infection in preweaned calves^24,28,31,36,37,39–43^. Certainly, there are some inconsistencies in the ages of preweaned and postweaned calves, or the age of the sampled cows is not clearly stated in previous studies. If calves aged < 6 months are defined as preweaned calves, the postweaned calves in this study will be classified as preweaned calves, but this is not consistent with the current situation of cattle farming in China. If this was the case, the prevalence of *G. duodenalis* in preweaned calves in this study was 29.3% (61/208), which is inconsistent with the higher *G. duodenalis* prevalence in preweaned calves (aged < 6 months) reported in the pooled global data^14^. As mentioned earlier, several factors influence the prevalence of *G. duodenalis* infection, age distribution, and diarrhea occurrence, such as the immature and susceptible immune system of younger animals^60^, asymptomatic immunocompetent individuals^14,62^, and the increased resistance due to nonspecific immunity acquired via breast milk^63^.

Overall, 146 plausible *SSU rRNA* sequences were identified as 13 assemblage A and 133 as assemblage E, with assemblage E being the dominant genotype. This finding is consistent with the studies reporting that assemblage E is the most common genotype in cattle worldwide^4,13,14,16–20^. Combining the sequence analysis results of 38 isolates of *gdh*, 47 isolates of *tpi*, and 70 isolates of *bg*, the genetic diversity of these positive *G. duodenalis* isolates was determined. The results revealed their genetic diversity, and seven isolates were identified to exhibit inconsistent assemblage. This finding is similar to the results reported previously in China and abroad^4,64^. It is also consistent with the results reported for 89,139 cattle from 48 countries in seven regions, with assemblage E being the most common, followed by assemblages A and A + E^14^. Transmission via environment (e.g., cyst contaminated water and food) may play a key role in parasite epidemiology^65^. Assemblages A and B are considered zoonotic^4,14,21–24^, and assemblage E has also been reported in humans in Egypt^66^, Brazil^67^, and Australia^68^. In China, cows are considered significant reservoirs of human giardiasis^69^. The results of the present study suggest that dairy cattle in Inner Mongolia pose a risk of causing *G. duodenalis* infection in humans.

Herein, the comparison of cattle belonging to different age groups for four of *G. duodenalis* assemblage genes revealed that preweaned calves only contained assemblage E, whereas postweaned calves contained assemblages E and A. Seven assemblage A + E were present in postweaned calves (n = 3), young cattle (n = 2), and adult cattle (n = 2). This finding is consistent with the report of preweaned calves containing only assemblage E in Sichuan, China^33^, and postweaned calves containing assemblages E, A, and A + E^34^. Furthermore, it is inconsistent with reports mentioning that assemblages E and A were detected in preweaned calves and postweaned calves in China^31,41^, USA^59,60^, and Europe^70^. A high prevalence of assemblage A in preweaned calves has also been reported^4^. Assemblage A infection has been reported in dairy cattle of all ages^59,60,71,72^, and assemblage E is also been reportedly common in adult cattle^47^. In addition, assemblage E is more common in calves than in adult cattle^73^. Herein, only assemblage E was found in the Hanggin Rear Banner field; all the other fields were found to have assemblages E and A, and three of them were also found to have assemblage A + E. In China, reports on cattle *G. duodenalis* assemblage varied with different sites. Assemblage E was only detected in Hubei Province^29^, Beijing^35^, Gansu^36,37^, Inner Mongolia^44^, and Qinghai^45^; assemblages E and A were detected in Shaanxi^31^, Jilin^28^, Jiangxi^43^, Tibet^46^, and Yunnan^47^; assemblages E and A + E were detected Hebei and Tianjin^40^; assemblages E, A, and A + E were detected in Xinjiang^4^, Liaoning^28^, Sichuan^33,34^, Guangdong^38,39^, Henan^41^, and Jiangsu^42^; assemblages E, B,^24,37^, and A were detected^37^ in Ningxia; assemblages E, A, B, and A + E were detected in Heilongjiang^26–28^ and Shanghai^32^; and assemblages E and D were detected in Taiwan^48^. However, the available data in China do not reflect the geographical distribution pattern of *G. duodenalis* assemblages.

Reports on genetic variations in *G. duodenalis* assemblage remain insufficient. The characteristics of individual loci of *G. duodenalis* often lead to inconsistent genotyping results^16^. The MLG model was used to better understand the diversity of human and animal *G. duodenalis* in different geographic regions, which can help reveal the potential and dynamic transmission of zoonosis^74^. Herein, 26 isolates containing the *gdh*, *tpi*, and *bg* were combined to obtain 25 different assemblage E MLGs with six subgroups. Consistent with the results of previous studies, numerous MLGs were identified in assemblage E. Additionally, *G. duodenalis* isolates that were classified in the same assemblage may be classified as distinct MLGs^4,31,32,34,37–39,41,75^.

The results of the present study suggest that dairy cattle in Inner Mongolia pose a risk of causing *G. duodenalis* infection in humans. In addition, *Giardia* are commonly found on fruits^76^, and vegetables^77,78^, and in various types of water^79–89^ in other regions of China. Therefore, further studies need to investigate the molecular epidemiology of cattle keepers and neighboring water sources in Inner Mongolia to evaluate the transmission dynamics of *G. duodenalis*, to adopt effective strategies to prevent and control *G. duodenalis* transmission among dairy cattle and humans in Inner Mongolia.

## Conclusions

To the best of our knowledge, the present study is the first to report *G*. *duodenalis* infection in dairy cattle in Inner Mongolia, thereby filling a gap in the molecular epidemiological data regarding giardiasis in Central Inner Mongolia. The results reconfirmed previous findings in other parts of China that *G. duodenalis* infection is common in dairy cattle. The livestock-specific *G*. *duodenalis* assemblage E was the dominant assemblage; however, zoonotic assemblage A was also present in Inner Mongolia. The distribution of bovine *G. duodenalis* assemblage E MLGs was diverse.

## Data Availability

All the sequences obtained in our laboratory have been uploaded to the GenBank database under the Accession Numbers OP189375 to OP189675.

## Abbreviations

MLG: Multilocus genotype
SSU rRNA: Small subunit ribosomal RNA
*gdh*: Glutamate dehydrogenase
*tpi*: Triosephosphate isomerase
*bg*: Beta-giardin
PCR: Polymerase chain reaction
CI: Confidence interval
OR: Odds ratio
